# Probiotics have beneficial metabolic effects in patients with type 2 diabetes mellitus: a meta-analysis of randomized clinical trials

**DOI:** 10.1038/s41598-020-68440-1

**Published:** 2020-07-16

**Authors:** Tícia Kocsis, Bálint Molnár, Dávid Németh, Péter Hegyi, Zsolt Szakács, Alexandra Bálint, András Garami, Alexandra Soós, Katalin Márta, Margit Solymár

**Affiliations:** 1 Institute for Translational Medicine, Medical School, University of Pécs, 12 Szigeti str., Pecs, 7624 Hungary; 2 Hungarian Academy of Sciences, University of Szeged, Momentum Gastroenterology Multidisciplinary Research Group, Szeged, Hungary; 3 Szentágothai Research Center, University of Pécs, Pecs, Hungary; 4 Heart Institute, Medical School, University of Pécs, Pecs, Hungary

**Keywords:** Diabetes, Dyslipidaemias

## Abstract

Probiotics have been reported to have a positive impact on the metabolic control of patients with type 2 diabetes. We aimed to systematically evaluate the effects of probiotics on cardiometabolic parameters in type 2 diabetes based on randomized controlled studies. MEDLINE, Embase, and CENTRAL databases were reviewed to search for randomized controlled trials that examined the effects of probiotic supplementation on cardiometabolic parameters in patients with type 2 diabetes. 32 trials provided results suitable to be included in the analysis. The effects of probiotics were calculated for the following parameters: BMI, total cholesterol levels, LDL, triglycerides, HDL, CRP, HbA1c levels, fasting plasma glucose, fasting insulin levels, systolic and diastolic blood pressure values. Data analysis showed a significant effect of probiotics on reducing total cholesterol, triglyceride levels, CRP, HbA1c, fasting plasma glucose, fasting insulin levels, and both systolic and diastolic blood pressure values. Supplementation with probiotics increased HDL levels however did not have a significant effect on BMI or LDL levels. Our data clearly suggest that probiotics could be a supplementary therapeutic approach in type 2 diabetes mellitus patients to improve dyslipidemia and to promote better metabolic control. According to our analysis, probiotic supplementation is beneficial in type 2 diabetes mellitus.

## Introduction

Type 2 diabetes mellitus is one of the major worldwide unresolved health challenges: it is a major risk factor for a number of common, sometimes potentially lethal diseases, such as hypertension, stroke, coronary heart disease^1^, or kidney failure and retinopathy^2^. According to the International Diabetes Federation, the worldwide prevalence of diabetes mellitus was 8.8% in 2015, and by 2040 the prevalence of diabetes in adults is predicted to rise to 10.4%^3^. The increasing prevalence of obesity provides ground to the rising prevalence of type 2 diabetes^4^. Even though the main cause of obesity is the imbalanced calorie intake, one intriguing hypothesis links the composition of the human gut microbiome to human energy homeostasis; for instance with their ability to promote adiposity through manipulation of host genes and metabolism, an altered microbiome can lead to predisposition to obesity^5^. The alteration in the gut microbiota has recently been recognized as a key environmental factor resulting in metabolic diseases, such as type 2 diabetes. In fact, the gut microbiota is involved in the maintenance of host energy homeostasis and in the stimulation of host immunity through a molecular crosstalk^6^.

Although many drugs have been developed to maintain glycemic control and normalize blood glucose levels either via enhanced insulin production and utilization, suppressed glucose production and absorption, by blocking urine glucose re-absorption and increasing glucose excretion in urine, or the combination of these^7^, these drugs may cause several adverse effects such as sulphonylureas carry a risk of causing acute severe hypoglycemia; lactic acidosis is also a potentially serious adverse effect associated with the use of biguanides; and gastrointestinal adverse effects may occur with the use of metformin^8^. Alternatively, the potential role of modifications in the gut microbiome had been explored as a new complementary therapeutic strategy^9^. Clinical evidence supports the hypothesis that the modulation of the gut microbiota by probiotics could be effective in prevention and management of diabetes^10,11^.

Probiotics are live microorganisms that, when administered in adequate amounts, confer a health benefit on the host. The healthy human body contains such microbes physiologically; and they can be obtained in forms of over-the-counter food supplements as well. Over the last few years, probiotics, especially the lactobacillus species were shown to be effective in the therapy of type 2 diabetes^12^. In type 2 diabetes, gut microbiome is found to be different from that in the healthy population. In a human study, the amount of *Firmicutes* bacteria was lower, whereas the number of *Bacteroides* and *Proteobacteria* is higher in the gastrointestinal tract of patients with type 2 diabetes compared to non-diabetic persons^13^. According to the study^13^, the ratio of *Bacteriodes* and *Firmicutes* species had positive correlation with decreased insulin resistance, however, causality has not been proven yet. Following innovative dietary strategies, it seems possible to maintain euglycemia by normalizing the altered microbiome, and to prevent long-term micro- and macrovascular complications of type 2 diabetes^9^. Although, there have been numerous bacterial species investigated in the therapy of type 2 diabetes, no consensus has been obtained regarding the effectivity and the most effective species. For instance, an earlier meta-analysis suggested, that the intake of certain *Lactobacillus* species, such as *L. fermentum, L. ingluviei* and *L. acidophilus* can lead to weight gain, while the ingestion of *L. gasseri* and *L. plantarum* might end up in weight loss both in animal and human studies^14^. Previous meta-analysis in this field were not conducted with assessment of the evidence quality levels and the number of identified trials that met their inclusion criteria was relatively low (7–12 trials)^15–19^. Two meta-analysis found no significant effects of probiotics on lipid profile^16,19^ and two meta-analysis found decreased indexes of lipid profiles^17,18^. These contradictory reports on the effect of probiotics inspired us to conduct an updated meta-analysis to assess the effect of probiotic therapies in diabetes mellitus type 2 exclusively from randomized controlled trials.

## Materials and methods

### Protocol and registration

This meta-analysis was reported according to the recommendation of the Preferred Reporting Items for Systematic Reviews and Meta-Analysis (PRISMA) guidelines^20^. Pre-specified protocol of this meta-analysis was published in the Prospero Center for Reviews and Dissemination (PROSPERO) under the registration number of CRD42019137997.

### Search strategy

Meta-analysis was performed using the PICO format: whether an intervention with probiotic supplementation (I) compared with placebo (C) has any effect on metabolic parameters (body mass index (BMI), total-cholesterol, low density lipoprotein (LDL), triglycerides (TG), high density lipoprotein (HDL), high sensitivity C-reactive protein (hs-CRP), haemoglobin A1c (HbA1c), fasting plasma glucose and insulin levels, systolic and diastolic blood pressure (SBP, DBP) (O) in patients with diabetes mellitus type 2 (P). In general, the following search terms were used in all databases: diabetes mellitus type 2 AND (probiotic* OR lactobacillus OR saccharomyces OR enterococcus OR escherichia coli OR streptococcus OR bifidobacterium) AND random*. Trials were identified by searching MEDLINE (via PubMed), EMBASE and CENTRAL databases up to 5th of April 2019. No filters or restrictions were applied. We included human trials without any restriction to language or year of publication.

### Eligibility criteria and study selection

Duplicates were removed by the EndNote software first automatically, then manually. Randomized controlled trials in which probiotics in the form of any pharmaceutical formulations or dairy products administered to adult patients with type 2 diabetes were included after title and abstract screening. Combination therapy was not an exclusion criterion. Subsequently, full texts of the articles were reviewed for inclusion of eligible studies. Two review authors (TK and BM) selected the articles fulfilling the inclusion criteria independently, and any disagreement was resolved by consensus.

### Data collection

At the end of the screening process, relevant data were independently extracted from studies by the two review authors and any disagreement was resolved by consensus. Data were extracted into a standardized excel sheet form. Data extracted from the papers included: number of participants, dosage, the intervention used, study duration and the outcome parameters including BMI changes as primary outcome and changes in the total-cholesterol, LDL, TG, HDL, hs-CRP, HbA1C, fasting plasma glucose and insulin levels, SBP and DBP as secondary outcomes. The authors of the studies and year of publication were also recorded. Mean values for control and intervention groups, along with the measure of dispersion were extracted.

### Risk of bias assessment

Two review authors assessed the risk of bias of the studies independently, and any disagreement was resolved by consensus. The assessment was performed using the updated version of the Cochrane risk-of-bias tool for randomized trials (RoB 2) with the following domains: bias arising from the randomization process, bias due to deviations from intended interventions, bias due to missing outcome data, bias in measurement of the outcome, bias in selection of the reported result, and overall bias^21^.

### Quality of evidence

We used the Grading of Recommendations, Assessment, Development and Evaluation (GRADE) approach to rate the quality of evidence on our primary outcomes.

### Statistical analysis

We calculated weighted mean differences (WMD) with 95% confidence intervals (CI) as effect size data based on the difference of before-after values in the intervention and comparator groups. Means were compared by assessing the overlap of CIs. Between-study heterogeneity was tested with (a) chi2 statistics (where *p* < 0.1 was considered significant) and (b) I^2^ statistics, where 75–100% was considered considerable^22^. Due to the methodological differences between interventions, we performed all analysis under the random effect assumption. To assess small study effect, we used visual inspection of funnel plots and Egger's test was performed. If *p* ≥ 0.1, publication bias is unlikely to occur in the sample. We used trial sequential analysis to investigate if alpha and beta-type errors affect our estimates. All analyses were performed with the Comprehensive Meta-analysis software (Biostat, Inc., Engelwood, MJ, USA) and Stata 11 SE (Stata Corp) software.

## Results

### Characteristics of the included studies

**Figure 1:**
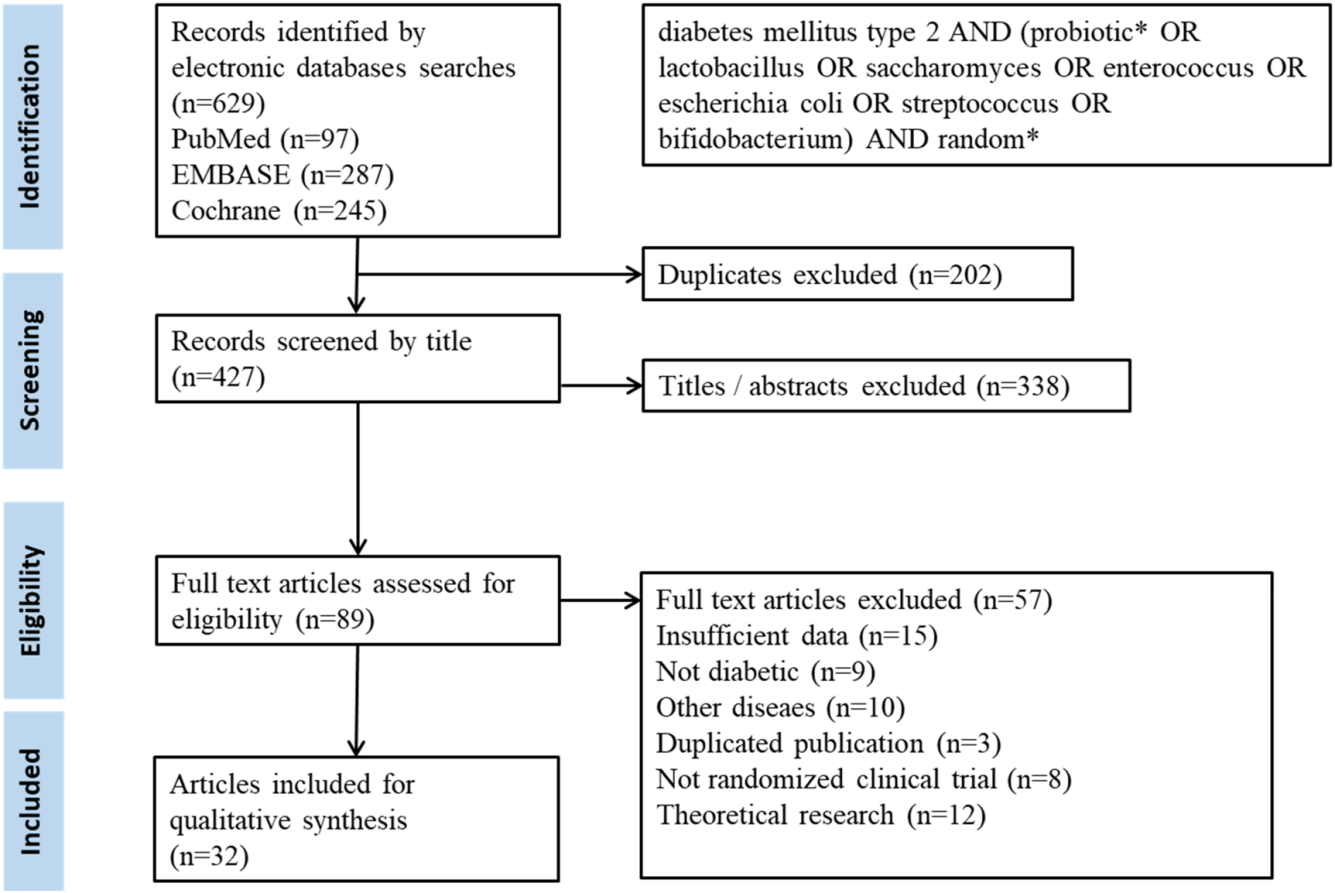
A flow diagram detailing process of study selection for the meta-analysis.

A flow chart of selection for the meta-analysis is shown in Fig. 1.

32 eligible studies were included in the meta-analysis^12,23–52^. Main characteristics of the studies included are shown in Table 1. Fifteen studies administered one bacterial species, while the rest of the studies used a combination of more than one strain: seventeen studies administered two to seven bacterial species. In one article, the flora of the probiotic yoghurt of the intervention group was enriched with specific strains, however placebo yoghurt also contained bacterial flora^12^. In three articles, probiotics were co-administered with chromium^35,51, 52^, in one article with selenium^42^, and in one article with vitamin D^43^. The duration of intervention ranged from four to 34 weeks. Seventeen of the 32 articles were published from Iran, two from Saudi Arabia, two from Ukraine, two from Brazil, two from India, and the remaining ones from Malaysia, Denmark, Taiwan, Poland, Sweden, Japan, and Greek.

**Table 1:** Characteristics and findings of the studies included in the analysis.

	Country	Total number of participants	Type of participants	Strains used	Daily dose	Duration of treatment (weeks)	Outcomes
Abbasi et al.^23^	Iran	40	T2DM (for: > 1 year, FPG: > 126 mg/dL, PPG: > 200 mg/dL, microalbuminuria, GFR: > 60 mL/min)	*L. plantarum*	2 × 10^7^ CFU/mL	8	BW, BMI, TC, TG, LDL-C, HDL-C
Asemi et al.^24^	Iran	54	T2DM (FPG: > 126 mg/dL/PPG: > 200 mg/dL/HbA1c: > 6, 5%)	*L. acidophilus* *L. casei* *L. rhamnosus* *L. bulgaricus* *B. brevei* *B. longum* *Strep. thermophilus*	2 × 10^9^ CFU 7 × 10^9^ CFU 1.5 × 10^9^ CFU 2 × 10^8^ CFU 2 × 10^10^ CFU 7 × 10^9^ CFU 1.5 × 10^9^ CFU	8	BW, BMI, TC, TG, LDL-C, HDL-C, CRP, HbA1c, FPG, Insulin
Bahmani et al.^25^	Iran	81	T2DM (FPG: > 126 mg/dL/PPG: > 200 mg/dL/HbA1c: > 6,5%)	*L. sporogenes*	3 × (40 × 10^8^ CFU)	8	BW, BMI
Bayat et al.^26^	Iran	80	T2DM (FPG: > 126 mg/dL and controlled lipid profile w/o changing the drug instruction)	*Not specified, probiotic yoghurt*	Not known	8	TC, TG, LDL-C, HDL-C, CRP, HbA1c, FPG
Ejtahed et al.^12^	Iran	60	T2DM (for: > 1 year and BMI: < 35 and LDL-C: > 2.6 mmol/L )	*L. acidophilus* *B. lactis* *L. bulgaricus* *Strep. thermophilus*	300 × (1.05 × 10^6^ CFU) 300 × (1,19 × 10^6^ CFU) Not known Not known	6	TC, TG, LDL-C, HDL-C
Feizzollahzadeh et al.^27^	Iran	40	T2DM	*L. plantarum*	2 × 10^7^ CFU	8	TG, LDL-C, HDL-C, CRP, FPG
Firouzi et al.^28^	Malaysia	136	T2DM (for: > 0.5 year, HbA1c: 6.5–12%, FPG: < 15 mmol/L, BMI: 18.5–40)	*L. acidophilus* *L. casei* *L. lactis* *B. bifidum* *B. longum* *B. infantis*	10^10^ CFU 10^10^ CFU 10^10^ CFU 10^10^ CFU 10^10^ CFU 10^10^ CFU	12	BW, BMI, TC, TG, LDL, HDL, CRP, HbA1c, FPG, Insulin, SBP, DBP
Hariri et al.^29^	Iran	40	T2DM (for: > 1 year, FPG: > 126 mg/dL, PPG: > 200 mg/dL)	*L. plantarum*	200 × (2 × 10^7^ CFU)	8	BW, BMI, SBP, DBP
Hove et al.^30^	Denmark	41	T2DM (for: > 1 year, HbA1c: 6–10%)	*L. helveticus*	300 mL	12	BW, BMI, TC, TG, LDL-C, HDL-C, CRP, HbA1c, FPG
Hsieh et al.^31^	Taiwan	68	T2DM (for: 0.5 years, BMI: > 18, HbA1c: 7–10%)	*L. routeri (live)* *L. routeri (heat killed)*	2 × (2 × 10^9^ CFU) 2 × (1 × 10^10^ CFU)	12	TC, TG, LDL-C, HDL-C, HbA1c, Insulin, SBP, DBP
Khalili et al.^32^	Iran	40	T2DM (for: > 1 years, BMI: < 35)	*L. casei*	10^8^ CFU	8	BW, BMI, HbA1c, FPG, Insulin, SBP, DBP
Kobyliak et al.^33^	Ukraine	58	T2DM (BMI: > 25, NAFLD)	*Lactococcus, Bifidobacterium, Propionibacterium, Acetobacter*	10 × (6 × 10^10^ CFU) 10 × (1 × 10^10^ CFU) 10 × (3 × 10^10^ CFU) 10 × (3 × 10^6^ CFU)	8	TC, TG, LDL-C, HDL-C
Kobyliak et al.^34^	Ukraine	53	T2DM (for: 0.5 years, BMI: > 25, HbA1c: 6.5–11%, HOMA-IR: > 2)	*Lactococcus, Bifidobacterium, Propionibacterium, Acetobacter*	10 × (6 × 10^10^ CFU) 10 × (1 × 10^10^ CFU) 10 × (3 × 10^10^ CFU) 10 × (3 × 10^6^ CFU)	8	BW, BMI, HbA1c, FPG, Inulin
Król et al.^35^	Poland	20	T2DM (BMI: 35.3 (9.2), HbA1c: > 7.0%)	*Saccharomyces cerevisiae*	5 × 100 μg	8	BMI, TC, TG, LDL-C, HDL-C, HbA1c, FPG, Insulin
Mafi et al.^36^	Iran	60	T2DM with diabetic nephropathy (Proteinuria: > 0.3 g/day)	*L. acidophilus* *L. reuteri* *L. phermentum* *B. bifidum*	2 × 10^9^ CFU 2 × 10^9^ CFU 2 × 10^9^ CFU 2 × 10^9^ CFU	12	BW, BMI, TC, TG, LDL-C, HDL-C, CRP, HbA1c, FPG, Insulin
Mazloom et al.^37^	Iran	34	T2DM (for: < 15 years, FPG: > 126 mg/dL)	*L. acidophilus* *L. bulgaricus* *L, bifidum* *L. casei*	Not known	6	TC, TG, LDL-C, HDL-C, CRP, FPG
Mobini et al.^38^	Sweden	44	T2DM (for: > 0.5 years, waist: > 80 cm [F] or > 94 cm [M], HbA1c: 6.7–10.4%, BMI: 25–45)	*L. reuteri* *L. reuteri*	10^8^ CFU 10^10^ CFU	12	BW, BMI, TC, TG, LDL-C, HDL-C, CRP, HbA1c, FPG, SBP, DBP
Mohamadshai et al.^39^	Iran	44	T2DM (BMI: > 25)	*L. bulgaricus* *Strep. thermophilus* *B. lactis* *L. acidophilus*	Not known Not known 300 × (3,7 × 10^6^ CFU) 300 × (3,7 × 10^6^ CFU)	8	BW, BMI, CRP, HbA1c, FPG
Moroti et al.^40^	Brazil	20	T2DM (TC: > 200 mg/dL, TG: > 150 mg/dL, FPG: > 110 mg/dL)	*L. acidophilus* *B. bifidum*	200 × (1 × 10^8^ CFU) 200 × (1 × 10^8^ CFU)	4,3	TC, TG, HDL-C, FPG
Ostadrahimi et al.^41^	Iran	60	T2DM (for: < 20 years, FPG: > 125 mg/dL)	*Strep. thermophiles* *L. casei* *L. acidophilus* *B. lactis*	Not known 1,200 × (15 × 10^6^ CFU) 1,200 × (25 × 10^6^ CFU) 1,200 × (8 × 10^6^ CFU)	8	BW, TC, TG, LDL-C, HDL-C, HbA1c, FPG
Raygan et al.^42^	Iran	54	T2DM w/ 2- or 3-vessel CHD	*L. acidophilus* *L. reuteri* *L. fermentum* *B. bifidum*	2 × 10^9^ CFU/g 2 × 10^9^ CFU/g 2 × 10^9^ CFU/g 2 × 10^9^ CFU/g	12	BW, BMI, TC, TG, LDL-C, HDL-C, CRP, FPG, Insulin, SBP, DBP
Raygan et al.^43^	Iran	60	T2DM w/ 2- or 3-vessel CHD	*L. acidophilus* *L. reuteri* *L. fermentum* *B. bifidum*	2 × 10^9^ CFU/g 2 × 10^9^ CFU/g 2 × 10^9^ CFU/g 2 × 10^9^ CFU/g	12	BW, BMI, TC, TG, LDL-C, HDL-C, CRP, FPG, Insulin, SBP, DBP
Raygan et al.^44^	Iran	60	T2DM w/ 2- or 3-vessel CHD	*B. bifidum* *L. casei* *L. acidophilus,*	2 × 10^9^ CFU/g 2 × 10^9^ CFU/g 2 × 10^9^ CFU/g	12	BW, BMI, TC, TG, LDL-C, HDL-C, CRP, FPG, Insulin, SBP, DBP
Sabico et al.^45^	Saudi-Arabia	78	T2DM (for: < 0.5-year, w/o complications, HbA1c: < 7%)	*B. bifidum* *B. lactis* *L. acidophilus* *L. brevis* *L. casei* *L. salivarius* *Lactococcus lactis W19* *Lactococcus lactis W58*	2 × (2.5 × 10^9^ CFU/g)	12	BW, BMI, TC, TG, LDL-C, HDL-C, FPG, Insulin, SBP, DBP
Sabico et al.^45^	Saudi-Arabia	61	T2DM (for: < 0.5-year, w/o complications, HbA1c: < 7%)	*B. bifidum* *B. lactis* *L. acidophilus* *L. brevis* *L. casei* *L. salivarius* *Lactococcus lactis W19* *Lactococcus lactis W58*	2 × (2.5 × 10^9^ CFU/g)	34,13	BMI, TC, TG, LDL-C, HDL-C, CRP, FPG, Insulin, SBP, DBP
Sato et al.^46^	Japan	68	T2DM (HbA1c: 6–8%)	*L. casei*	4 × 10^10^ CFU	16	BMI, TC, TG, HDL-C, CRP, HbA1c, FPG
Shakeri et al.^47^	Iran	52	T2DM (FPG: > 126 mg/dL/PPG: > 200 mg/dL/HbA1c: > 6,5%)	*L. sporogenes*	3 × (40 × 10^8^ CFU)	8	BW, BMI, TC, TG, LDL-C, HDL-C, FPG,
Sharma et al.^48^	India	40	T2DM (newly onset)	*Saccharomyces cerevisiae*	9 g	12	BMI, TC, TG, LDL-C, HDL-C, HbA1c, FPG, SBP, DBP
Sheth et al.^49^	India	35	T2DM (pre-hypertensive)	*Lactobacillus* *Bifidobacterium* *Streptococcus*	Not known Not known Not known	6.43	HbA1c, FPG
Tajadadi-Ebrahimi et al. 2014	Iran	71	T2DM (FPG: > 126 mg/dL/PPG: > 200 mg/dL/HbA1c: > 6.5%)	*L. sporogenes,*	3 × (40 × 10^8^ CFU)	8	BW, BMI, CRP, FPG, Insulin
Tonucci et al.^50^	Brazil	55	T2DM (for: > 1 year, BMI: < 35)	*Strep thermophilus* *L. acidophilus* *B. lactis*	Not known 120 × 10^9^ CFU 120 × 10^9^ CFU	6	TC, TG, LDL-C, HDL-C, HbA1c, FPG, Insulin
Yanni et al.^51^	Greece	30	T2DM (for: > 1 year, BMI: < 31, FPG: > 125 mg/dL, HbA1c: < 8.5%)	*Saccharomyces cerevisiae*	Not known	12	BW, BMI, TC, TG, HDL-C, CRP, HbA1c, FPG, Insulin, SBP, DBP

*T2DM* type 2 diabetes mellitus, *FPG* fasting plasma glucose, *PPG* postprandial plasma glucose, *HbA1c* glycated hemoglobin, *GFR* glomerular filtration rate, *BW* body weight, *BMI* body mass index, *TC* total cholesterol, *TG* triglyceride, *LDL-C* low-density lipoprotein, *HDL-C* high-density lipoprotein, *CFU* colony forming unit, *CRP *C-reactive protein, *SBP* systolic blood pressure, *DBP* diastolic blood pressure, *L. *Lactobacillus, *B.* Bifidobacterium, *Strep.* Streptococcus, *NAFLD* non-alcoholic fatty liver disease, *CHD* coronary heart disease, *HOMA-IR* Homeostatic Model Assessment for Insulin Resistance.

### Summary of findings

Data of outcome parameters are summarized in Table 2.

**Table 2:** Summary data of outcome parameters.

	N	WMD	CI low	CI high	*p*	I^2^ (%)	p (I^2^)
BMI (kg/m^2^)	17	− 0.17	− 0.38	0.04	0.114	86.6	< 0.001
T-chol (mg/dL)	21	− 10.06	− 15.94	− 4.18	**0.001**	93.2	< 0.001
LDL (mg/dL)	20	− 3.77	− 8.47	0.93	0.116	88.6	< 0.001
TG (mg/dL)	21	− 17.18	− 26.17	− 8.19	** < 0.001**	34.0	0.065
HDL (mg/dL)	22	1.62	0.21	3.04	** < 0.001**	57.4	< 0.001
CRP (mg/dL)	16	− 0.43	− 0.8	− 0.07	**0.019**	64.3	< 0.001
HbA1c (%)	14	− 0.33	− 0.53	− 0.13	**0.001**	75.9	< 0.001
FPG (mg/dL)	24	− 16.52	− 23.28	− 9.76	** < 0.001**	66.2	< 0.001
Insulin (µIU/mL)	15	− 1.40	− 2.52	− 0.27	**0.015**	46.8	0.024
SBP (mmHg)	14	− 1.79	− 3.09	− 0.49	**0.007**	0.0	0.89
DBP (mmHg)	14	− 1.32	− 2.42	− 0.21	**0.019**	0.0	0.838

Bold values indicate statistically significant weighted mean differences between the intervention and control groups, where *p* < 0.05.

*N* number of RCTs, *WMD* weighted mean difference, *CI* confidence interval, *BMI* body mass index, *T-chol* total cholesterol, *LDL* low-density lipoprotein, *TG* triglyceride, *HDL* high-density lipoprotein, *CRP* C-reactive protein, *HbA1c* hemoglobin A1c, *FPG* fasting plasma glucose, *SBP* systolic blood pressure, *DBP* diastolic blood pressure.

The summary of findings table provides a synopsis of the analysis (Table 3).

**Table 3:** Probiotics consumption compared to control in diabetes mellitus type 2.

Certainty assessment							No. of patients		Effect	Certainty	Importance
№ of studies	Study design	Risk of bias	Inconsistency	Indirectness	Imprecision	Other considerations	Probiotics	Control	Absolute (95% CI)		
**Body mass index**											
17	Randomized trials	Not serious	Serious^a^	Very serious^b^	Serious ^c^	None	498	497	WMD 0.17 kg/m^2^ lower (0.38 lower to 0.04 higher)	Very low	Important
**Total cholestero**l											
21	Randomized trials	Not serious	Very serious^a^	Very serious^b^	Not serious	None	596	600	WMD 10.06 mg/dL lower (15.94 lower to 4.18 lower)	Very low	Important
**LDL**											
20	Randomized trials	Not serious	Serious^a^	Very serious^b^	Not serious	None	546	544	WMD 3.77 mg/dL lower (8.47 lower to 0.93 higher)	Very low	Important
**Triglyceride**											
21	Randomized trials	Not serious	Not serious	Very serious^b^	Not serious	None	546	548	WMD 17.18 mg/dL lower (26.17 lower to 8.19 lower)	Low	Important
**HDL**											
22	Randomized trials	Not serious	Not serious	Very serious^b^	Serious ^d^	None	594	598	WMD 1.62 mg/dL higher (0.21 higher to 3.04 higher)	Very low	Important
**CRP**											
16	Randomized trials	Not serious	Serious ^e^	Very serious^b^	Serious ^d^	None	467	470	WMD 0.43 mg/l lower (0.8 lower to 0.07 lower)	Very low	Important
**HbA1c**											
14	Randomized trials	Not serious	Serious^a^	Very serious^b^	Not serious	None	395	372	WMD 0.33% lower (0.53 lower to 0.13 lower)	Very low	Important
**Fasting plasma glucose**											
24	Randomized trials	Not serious	SERIOUS^a^	Very serious^b^	Not serious	NONE	649	627	WMD 16.52 mg/dL lower (23.28 lower to 9.76 lower)	Very low	Important
**Fasting insulin**											
15	Randomized trials	Not serious	NOT serious	Very serious^b^	Not serious	None	455	451	WMD 1.4 µIU/mL lower (2.52 lower to 0.27 lower)	Low	Important
**Systolic blood pressure**											
14	Randomized trials	Not serious	Not serious	Very serious^b^	Not serious	Publication bias strongly suspected ^f^	417	418	WMD 1.79 Hgmm lower (3.09 lower to 0.49 lower)	Very low	Important
**Diastolic blood pressure**											
14	Randomized trials	Not serious	Not serious	Very serious^b^	Not serious	Publication bias strongly suspected ^f^	417	418	WMD 1.32 Hgmm lower (2.42 lower to 0.21 lower)	Very low	Important

*CI* confidence interval.

^a^Considerable heterogeneity was detected.

^B^Differences between interventions were substantial.

^c^Unusually high confidence interval in two of the studies.

^d^Unusually high confidence interval in one of the studies.

^e^Moderate heterogeneity was detected.

^f^Egger's test was significant.

### Risk of bias within the individual studies

**Figure 2:**
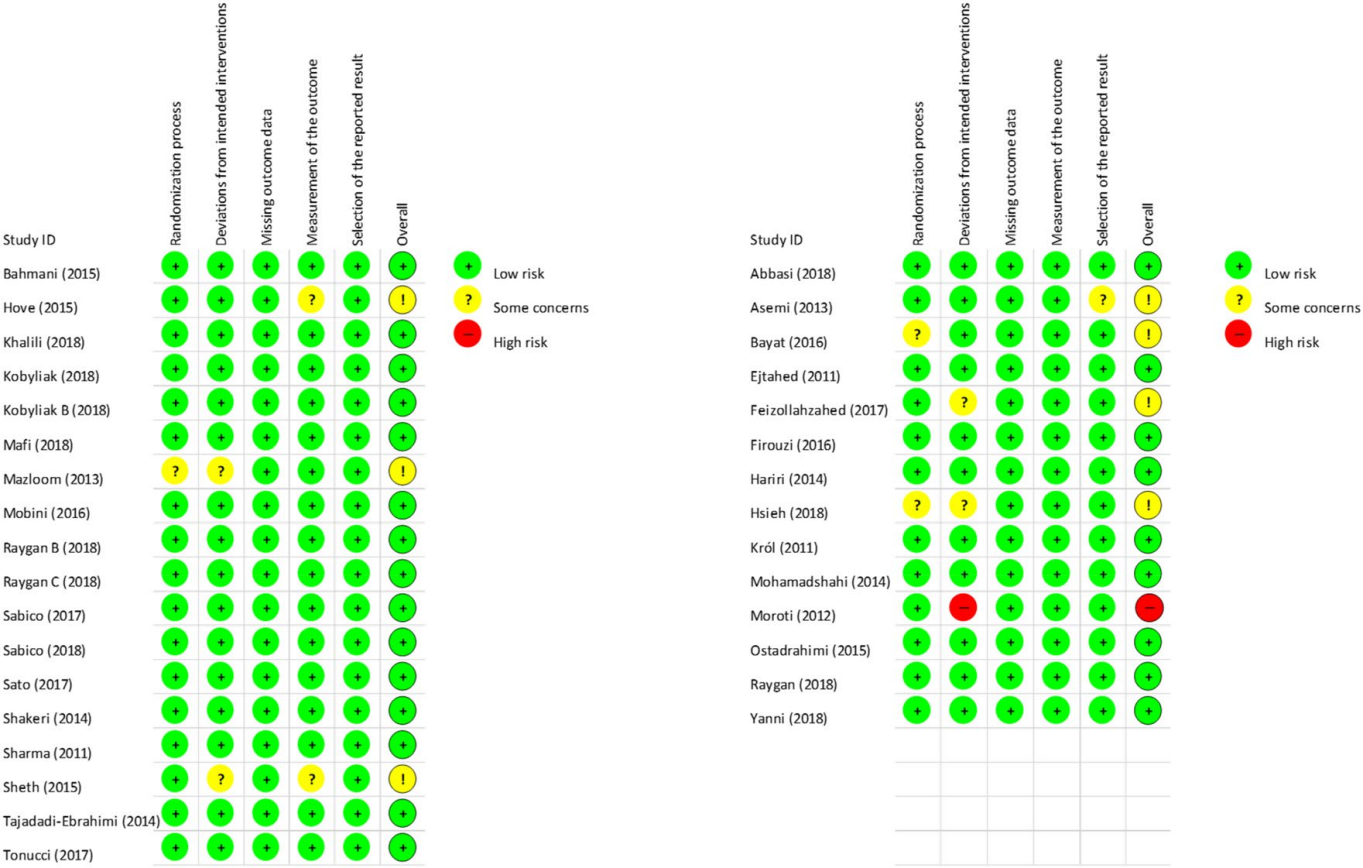
Risk of bias summary assessment of the included studies.

One study had high risk overall^40^. In seven studies, some concerns were detected; however, we found no articles with any concern about missing outcome data. The quality of the included studies is shown in detail in Fig. 2. Generally, the quality of the studies was good, in most cases with published pre-study protocols. We found three studies that were single-blind^37,51,52^, three more studies without blinding^26,46,48^, all the other articles contained double-blind studies.

### Probiotics did not change body mass index

Seventeen studies reported BMI changes. Pooled data showed no difference between the probiotic and placebo group. Considerable heterogeneity (I^2^: 86.6%, *p* < 0.001) was detected.

### Probiotics improved plasma lipid profile

**Figure 3:**
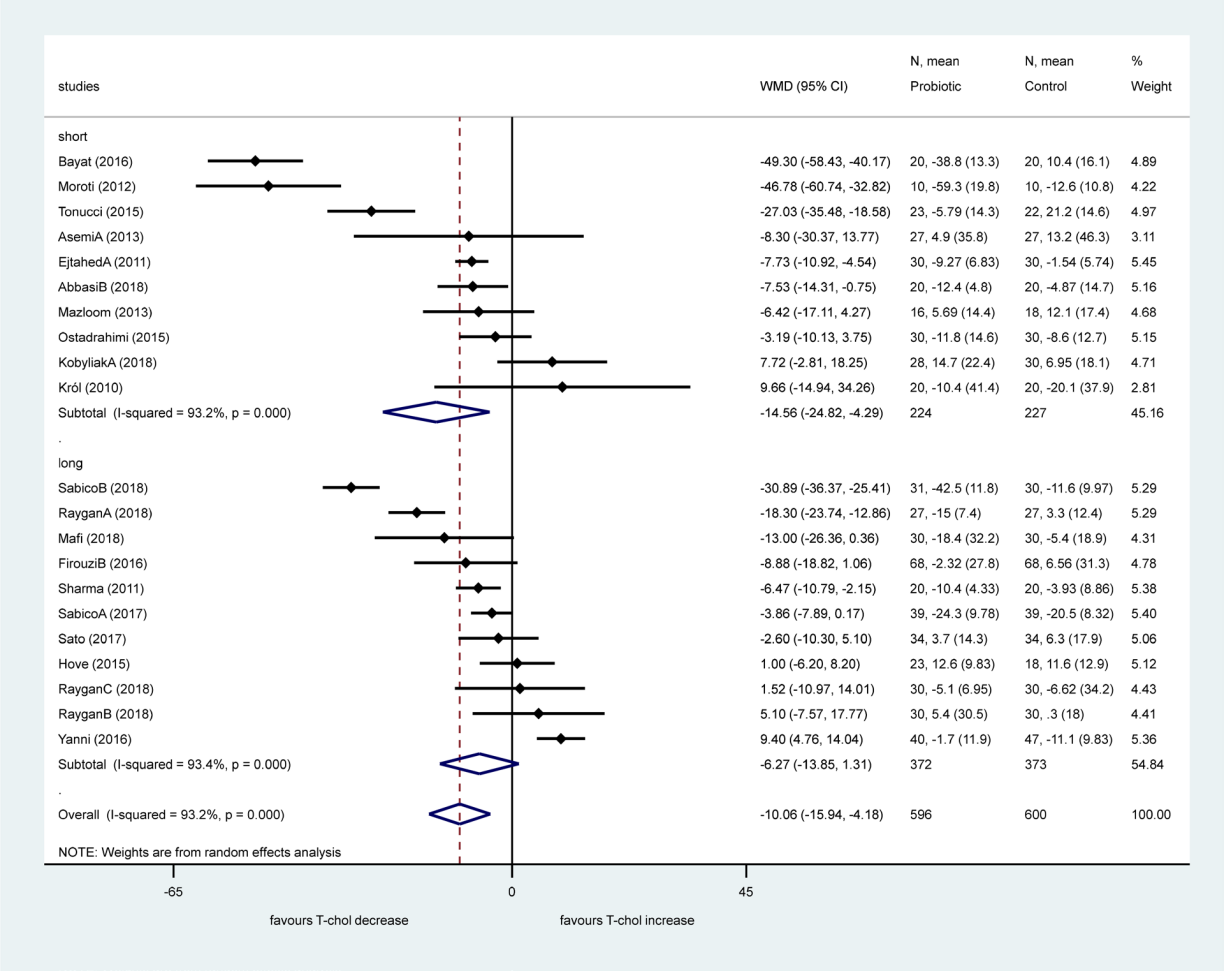
Forest plot for the effect of probiotics on total cholesterol (T-chol) compared to controls in pooled analysis. The shaded diamonds indicate the effect of probiotics in a particular study (weighted difference in mean). The horizontal lines represent 95% confidence intervals (CIs). The big diamond data marker indicates the pooled effect. The figure shows the summary of studies overall and subdivided by length of intervention. “long”: 12 weeks or longer, “short”: 8 weeks or shorter.

Twenty-one studies included data about the effect of probiotics on total-cholesterol level. Pooled data showed a significant effect of probiotics on reducing total-cholesterol levels with a mean difference of 10.06 mg/dL (95% CI − 15.94, − 4.18, *p* = 0.001) with a considerable heterogeneity (I^2^: 93.2%, *p* < 0.001). Sub-group analysis according to the length of investigation (i.e. duration of treatment) did not reduce the heterogeneity (Fig. 3, short: I^2^: 93.2%, *p* < 0.001; long: I^2^: 93.4%, *p* < 0.001). Short studies with 8 weeks treatment or shorter showed significant decrease of total cholesterol level (− 14.56 mg/dL, 95% CI − 24.82, − 4.29, *p* = 0.005), while studies of 12 weeks or longer showed no significant change (*p* = 0.105).

**Figure 4:**
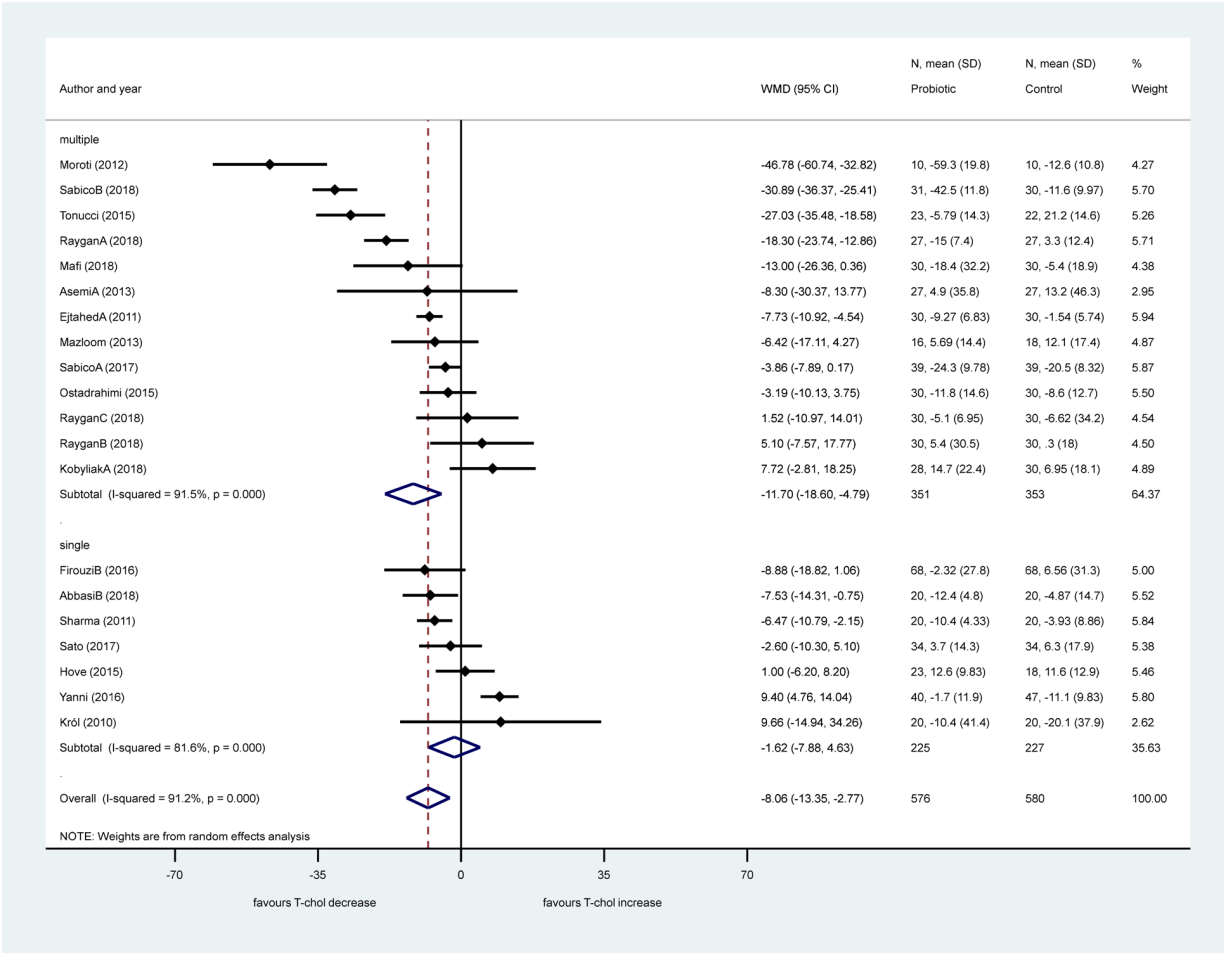
Forest plot for the effect of probiotics on total cholesterol (T-chol) compared to controls in pooled analysis. The shaded diamonds indicate the effect of probiotics in a particular study (weighted difference in mean). The horizontal lines represent 95% confidence intervals (CIs). The big diamond data marker indicates the pooled effect. The figure shows the summary of studies overall and subdivided by the number of bacterial species used. “multiple”: combination of bacteria, “single”: one bacterial species used.

We found a significant difference between these two sub-groups (*p* = 0.001). Sub-group analysis according to the number of bacterial strains (single or multiple, Fig. 4) did not change heterogeneity, either (multiple: I^2^: 91.5%, *p* < 0.001, single: I^2^: 81.6%, *p* < 0.001). The beneficial effect of multiple strains probiotics on total cholesterol was significant (− 11.70 mg/dL, 95% CI − 18.60, − 4.79, *p* = 0.001), however no difference was observed in single bacteria probiotic sub-group (*p* = 0.611) with significant difference between the two sub-groups (*p* < 0.001).

If we excluded the six articles where probiotics were co-supplemented with either vitamin D or chromium or selenium, and the article where the placebo group also got yoghurt with some bacteria, the heterogeneity did not change, nor the direction of the association (Figure S1).

Twenty studies reported data about LDL levels. No significant difference in LDL levels was observed between probiotic and placebo users (− 3.77 mg/dL, 95% CI − 8.47, 0.93, *p* = 0.116) with a considerable heterogeneity (I^2^: 88.6%, *p* < 0.001). Sub-group analysis according to the length of treatment (Figure S2) did not decrease the heterogeneity (short: I^2^: 88.9%, *p* < 0.001, long: I^2^: 89.5%, *p* < 0.001). Pooled studies with 8 weeks treatment period or shorter (*p* = 0.167) and studies of 12 weeks or longer showed no change of total cholesterol level (*p* = 0.493). We found no significant difference between these two groups (*p* = 0.555). Sub-group analysis according to the number of bacteria used (single or multiple, Figure S3 did not change heterogeneity, either (multiple: I^2^: 90.6%, *p* < 0.001, single: I^2^: 86.0%, *p* < 0.001). We found no effect of multiple-strain probiotics on total cholesterol (*p* = 0.139), and no difference was observed in single bacterium containing probiotic sub-group (*p* = 0.985), while there was no difference between the two sub-groups (*p* = 0.119). If we excluded the six articles where probiotics were co-supplemented with either vitamin D or chromium or selenium, and the article where the placebo group also got yoghurt with some bacteria, heterogeneity did not change, either (Figure S4). However, based on our trial sequential analysis, a number of 3,442 observations would be needed to provide sufficient statistical power (vs. the 1,090 patients in the current analysis) (Figure S5).

The meta-analysis of twenty-one trials showed a significant reduction of triglyceride by 17.18 mg/dL (95% CI − 26.17, − 8.19, *p* < 0.001). Heterogeneity was not substantial (34%, *p* = 0.065), sub-group analysis was therefore not conducted.

The meta-analysis of twenty-two trials showed a significant increase of HDL by 1.62 mg/dL (95% CI 0.21, 3.04, *p* = 0.025). I^2^ test (57.4%, *p* < 0.001) may represent moderate heterogeneity due to the differences between the interventions.

### Probiotics decreased CRP, HbA1c, fasting plasma glucose, fasting insulin, and blood pressure values

The meta-analysis of sixteen trials showed a significant decrease of CRP by 0.43 mg/dL (95% CI − 0.80, − 0.07, *p* = 0.019). I^2^ test (64.3%, *p* < 0.001) represented moderate heterogeneity.

Fourteen studies with reported the effect of probiotics on HbA1c levels. The decrease of HbA1c was slightly but significantly lower in the probiotic groups compared to placebo (− 0.33%, 95% CI − 0.53; − 0.13, *p* = 0.001). Heterogeneity was substantial (I^2^: 75.9%, *p* < 0.001).

**Figure 5:**
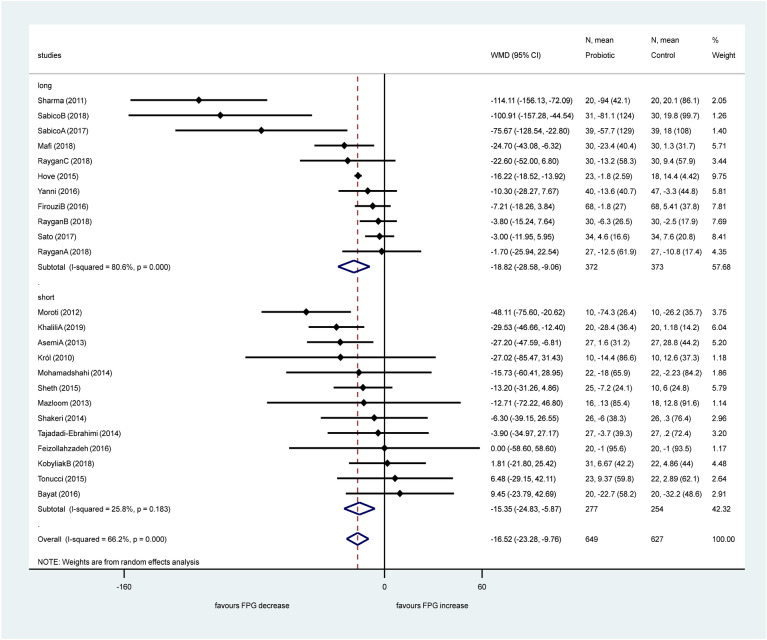
Forest plot for the effect of probiotics on fasting plasma glucose (FPG) compared to controls in pooled analysis. The shaded diamonds indicate the effect of probiotics in a particular study (weighted difference in mean). The horizontal lines represent 95% confidence intervals (CIs). The big diamond data marker indicates the pooled effect. The figure shows the summary of studies overall and subdivided by length of intervention. “long”: 12 weeks or longer, “short”: 8 weeks or shorter.

Twenty-four studies reported data about fasting plasma glucose. Pooled data showed a significant effect of probiotics in reducing fasting plasma glucose levels with a mean difference of − 16.52 mg/dL, (95% CI − 23.28; − 9.76, *p* < 0.001) with a substantial heterogeneity (I^2^: 66.2%, *p* < 0.001). Sub-group analysis according to the length of investigation (Fig. 5) did not change the heterogeneity in the long-term treatment sub-group (I^2^: 80.6, *p* < 0.001), however it decreased significantly in the short period therapy sub-group (I^2^: 25.8%, *p* = 0.183).

**Figure 6:**
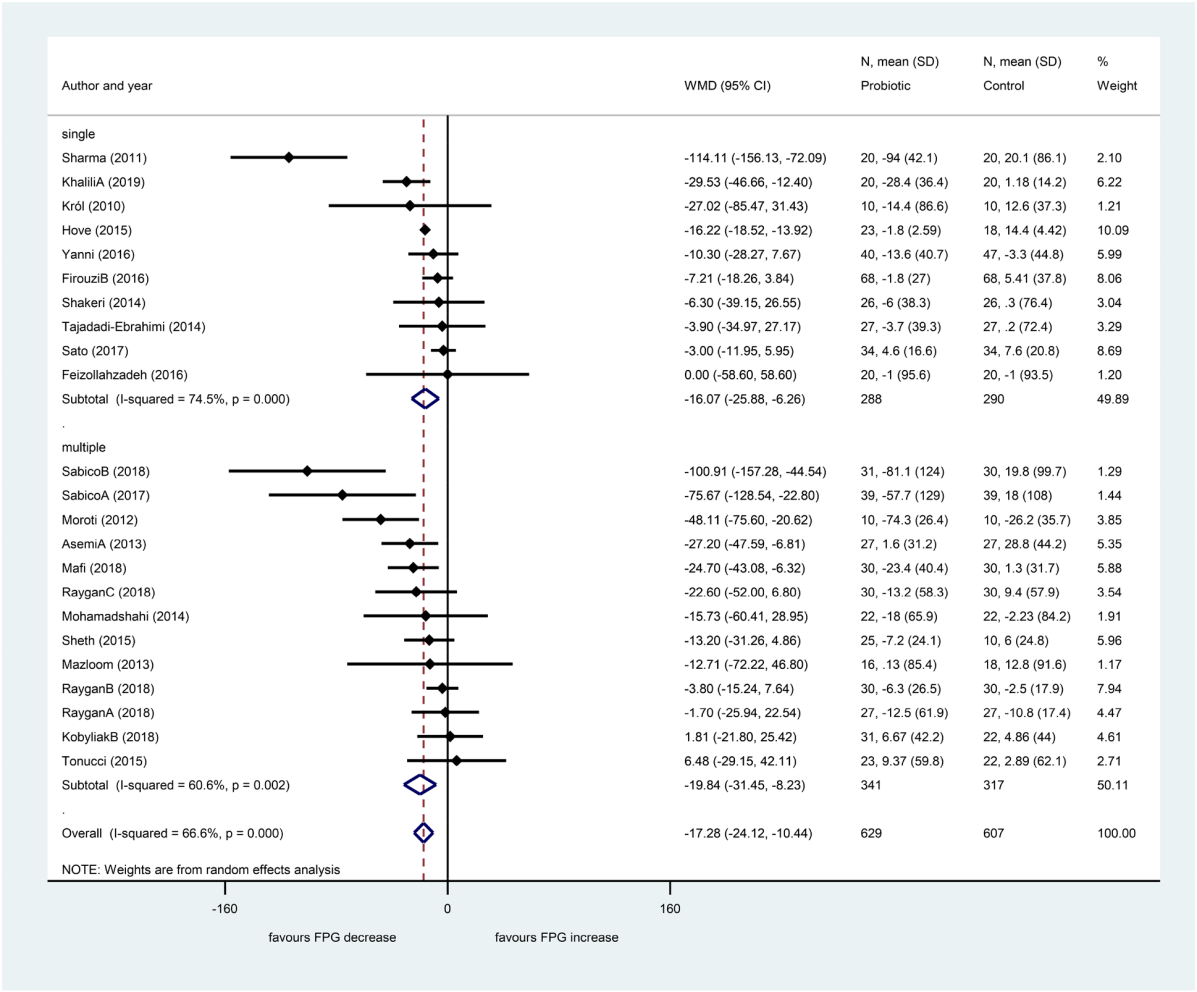
Forest plot for the effect of probiotics on fasting plasma glucose (FPG) compared to controls in pooled analysis. The shaded diamonds indicate the effect of probiotics in a particular study (weighted difference in mean). The horizontal lines represent 95% confidence intervals (CIs). The big diamond data marker indicates the pooled effect. The figure shows the summary of studies overall and subdivided by the number of bacterial species used. “multiple”: combination of bacteria, “single”: one bacterial species used.

Short studies with 8 weeks or shorter showed significant decrease of fasting plasma glucose level (− 15.35 mg/dL, 95% CI − 24.83, − 5.87, *p* = 0.002), and studies of 12 weeks or longer also showed a significant decrease (− 18.82 mg/dL, 95% CI − 28.58, − 9.06, *p* < 0.001). We found no significant difference between these two sub-groups (*p* = 0.723). Sub-group analysis according to the number of applied bacteria strains (single or multiple, Fig. 6) showed an increased heterogeneity in the single strain sub-group, and there was some minor decrease in the multiple strains sub-group (single: I^2^: 74.5%, *p* < 0.001; multiple: I^2^: 60.6%, *p* < 0.001).

The beneficial effect on fasting glucose level was significant both in the multiple strains probiotics subgroup (− 19.84 mg/dL, 95% CI − 31.45, − 8.23, *p* = 0.001) and in the single bacteria probiotic sub-group (− 16.07 mg/dL, 95% CI − 25.88, − 6.26, *p* = 0.001) with no significant difference between the two sub-groups (*p* = 0.892). If we excluded the six articles where probiotics were co-supplemented with either vitamin D or chromium or selenium, and the article where the placebo group also got yoghurt with some bacteria, the heterogeneity did not change, either (Figure S6).

The meta-analysis of fifteen trials showed a significant reduction of fasting insulin levels by 1.40 µIU/mL (95% CI − 2.52, − 0.27, *p* = 0.015). Heterogeneity was not significant (46.8%, *p* = 0.024), sub-group analysis was therefore not conducted.

Fourteen studies reported data about systolic and diastolic blood pressures. The meta-analysis showed a significant decrease both in systolic blood pressure (− 1.79 mmHg, 95% CI − 3.09; − 0.49, *p* = 0.007, I^2^: 0.0%, *p* = 0.890) and in diastolic blood pressure (− 1.32 mmHg, 95% CI − 2.42; − 0.21, *p* = 0.019, I^2^: 0.0%, *p* = 0.838). Since heterogeneity was not significant, no sub-group analysis was performed.

## Discussion

In the present meta-analysis, we aimed to evaluate the effects of probiotics on BMI and metabolic parameters in patients with type 2 diabetes mellitus. Data analysis showed a significant effect of probiotics in reduction of total cholesterol, triglyceride levels, CRP, HbA1c, fasting plasma glucose, fasting insulin levels and both systolic and diastolic blood pressure values. Supplementation with probiotics increased HDL levels however did not have a significant effect on BMI or LDL levels.

Such an evaluation is of high potential importance, as this patient group has especially high risk of cardiovascular diseases. It is crucial to reduce all the modifiable risk factors with efficient and multifactorial therapeutic methods and probiotic supplementation could be a complementary approach.

High total cholesterol levels, high blood pressure and type 2 diabetes mellitus are major risk factors of cardiovascular diseases. Reduction of the high total cholesterol and LDL levels in order to reduce the risk of major cardiovascular events is essential^53^. Every 1 mmol/L increment in total cholesterol levels increases the risk of cardiovascular diseases by 20% in women and by 24% in men^54^. Our results show that the consumption of probiotics has a decreasing effect on serum cholesterol levels. The mechanisms behind this reduction are that probiotics seem to be able to reduce serum cholesterol levels by reducing cholesterol absorption in the intestines^55^ and by the inhibition of HMG-CoA reductase enzyme thereby inhibiting endogenous cholesterol synthesis^56^.

The exact mechanism of action for the beneficial effects of probiotics on glycemia-related parameters is not fully elucidated. The favorable effects may be due to the immunoregulatory properties of probiotics. Cani et al. demonstrated, that metabolic endotoxemia dysregulates the inflammatory tone and triggers body weight gain and diabetes. Alterations in glucose homeostasis are associated with low-grade inflammation promoted by gut microbiota-derived lipopolysaccharide or endotoxin in mice^57^. Therefore, lowering plasma lipopolysaccharide concentration could be a strategy for the control of metabolic diseases, such as diabetes mellitus. Naito et al. showed that oral administration of Lactobacillus casei strain to obese mice led to a better insulin resistance through decreasing plasma levels of lipopolysaccharide-binding protein, a marker of endotoxemia^58^.

In our meta-analysis, probiotics significantly reduced total cholesterol, triglyceride levels, CRP levels, HbA1c levels, fasting plasma glucose levels, fasting insulin, and blood pressure together with the increase of the HDL levels. The observed small changes may not seem to be clinically significant, however the beneficial changes in many parameters can add up leading to a reduction in the severity of type 2 diabetes-related complications, and, as a consequence in lower mortality. The main strength of our study is that we included exclusively randomized clinical trials for evaluation and the number of the included trials were much higher than in other meta-analyses in this field. Some of our outcomes (triglyceride levels, systolic and diastolic blood pressure values) included a homogenous data set, so confounding factors are unlikely to distort our results. Waist to hip ratio was not measured in most of the articles, so that we could not pool the data.

We attempted to determine whether the observed heterogeneity in our outcomes was due to the differences in the length of treatment or in the number of probiotics used. However, according to our subgroup analyses high heterogeneity still remained unknown. We need more randomized clinical trials to be able to determine the most beneficial bacteria, the optimal dosage and treatment period. The identified significant heterogeneity is due to the significant differences between the intervention of the selected articles.

There are considerable limitations in our study. The diversified settings made it impossible to assess the effect of specific probiotic strains on the analyzed parameters. Many of the analyzed studies used probiotic mixtures or dairy products containing several probiotic strains. The data of diversity and richness of gut microbiota are absent in some of the included studies. The number of the probiotic species used in the included trials varied between the studies included in the analysis. The duration of probiotic intervention differed between the included trials. Consequently, substantial heterogeneity was observed between trials within this meta-analysis. No subgroup analysis was possible to assess which particular probiotic preparation could be the most effective to improve metabolic parameters in diabetic patients. Differences in population or differences in outcome were not considerable. The study aim was to test different cardiometabolic parameters in patients with diabetes mellitus type 2 in all included studies. However, differences in intervention were substantial, due to the fact, that different species or different probiotic combinations were used. This fact is worth to mention, because we are not able to have high quality evidence due to the very high indirectness.

In conclusion, according to our meta-analysis the administration of probiotics has a beneficial role in the management of type 2 diabetes regarding metabolic profile. We have shown a significant effect of probiotics in reducing total cholesterol, triglyceride levels, CRP, HbA1c, fasting plasma glucose, fasting insulin levels and both systolic and diastolic blood pressure values. Supplementation with probiotics increased HDL level and it did not had a significant effect on BMI or LDL levels. The practical implication of our study is that probiotic administration as a supportive intervention of type 2 diabetes could be incorporated into diabetes guidelines to beneficially modify cardiometabolic risk factors. Further studies are needed to investigate the combined effects of the different antidiabetic drugs and probiotic species.

## Supplementary information

Supplementary information.
