# Supplementary material for: Probiotics have beneficial metabolic effects in patients with type 2 diabetes mellitus: a meta-analysis of randomized clinical trials
Source: Sci Rep. 2020 Jul 16;10:11787. doi: 10.1038/s41598-020-68440-1 (PMC7366719; doi:10.1038/s41598-020-68440-1)
Supplement: Supplementary file 1 — Supplementary information. [file 41598_2020_68440_MOESM1_ESM.doc]

**Probiotics have beneficial metabolic effects in patients with type 2 diabetes mellitus: A meta-analysis of randomized clinical trials**

Tícia Kocsis,^1^ Bálint Molnár,^1^ Dávid Németh,^1^ Péter Hegyi,^1,2^ Zsolt Szakács,^1,3^ Alexandra Bálint,^1,4^ András Garami,^1^ Alexandra Soós,^1^ Katalin Márta,^1^ Margit Solymár,^1^*

^1^ Institute for Translational Medicine, Medical School, University of Pécs, Pécs, Hungary

^2^ Hungarian Academy of Sciences - University of Szeged, Momentum Gastroenterology Multidisciplinary Research Group, Szeged, Hungary

^3^ Szentágothai Research Center, University of Pécs, Pécs, Hungary

^4^ Heart Institute, Medical School, University of Pécs, Pécs, Hungary

**Short title:** Probiotic supplementation in type 2 diabetes mellitus

**Corresponding author:*

Margit Solymár, M.D., Ph.D.

Institute for Translational Medicine

Medical School, University of Pécs

12 Szigeti str., Pécs, Hungary H-7624

Phone: +36-72-536246

Fax: +36-72-536247

e-mail: [margit.solymar@aok.pte.hu](mailto:margit.solymar@aok.pte.hu)

| **Section/topic** | **#** | **Checklist item** | **Reported on page #** |
| --- | --- | --- | --- |
| **TITLE** | | |  |
| Title | 1 | Identify the report as a systematic review, meta-analysis, or both. | 1 |
| **ABSTRACT** | | |  |
| Structured summary | 2 | Provide a structured summary including, as applicable: background; objectives; data sources; study eligibility criteria, participants, and interventions; study appraisal and synthesis methods; results; limitations; conclusions and implications of key findings; systematic review registration number. | 2 |
| **INTRODUCTION** | | |  |
| Rationale | 3 | Describe the rationale for the review in the context of what is already known. | 3-4 |
| Objectives | 4 | Provide an explicit statement of questions being addressed with reference to participants, interventions, comparisons, outcomes, and study design (PICOS). | 5 |
| **METHODS** | | |  |
| Protocol and registration | 5 | Indicate if a review protocol exists, if and where it can be accessed (e.g., Web address), and, if available, provide registration information including registration number. | 5 |
| Eligibility criteria | 6 | Specify study characteristics (e.g., PICOS, length of follow-up) and report characteristics (e.g., years considered, language, publication status) used as criteria for eligibility, giving rationale. | 5 |
| Information sources | 7 | Describe all information sources (e.g., databases with dates of coverage, contact with study authors to identify additional studies) in the search and date last searched. | 5 |
| Search | 8 | Present full electronic search strategy for at least one database, including any limits used, such that it could be repeated. | 5. Supp. |
| Study selection | 9 | State the process for selecting studies (i.e., screening, eligibility, included in systematic review, and, if applicable, included in the meta-analysis). | 6, Fig1 |
| Data collection process | 10 | Describe method of data extraction from reports (e.g., piloted forms, independently, in duplicate) and any processes for obtaining and confirming data from investigators. | 6 |
| Data items | 11 | List and define all variables for which data were sought (e.g., PICOS, funding sources) and any assumptions and simplifications made. | 5-6 |
| Risk of bias in individual studies | 12 | Describe methods used for assessing risk of bias of individual studies (including specification of whether this was done at the study or outcome level), and how this information is to be used in any data synthesis. | 6, 8, Fig2 |
| Summary measures | 13 | State the principal summary measures (e.g., risk ratio, difference in means). | 7 |
| Synthesis of results | 14 | Describe the methods of handling data and combining results of studies, if done, including measures of consistency (e.g., I^2^) for each meta-analysis. | 7 |

| **Section/topic** | **#** | **Checklist item** | **Reported on page #** |
| --- | --- | --- | --- |
| Risk of bias across studies | 15 | Specify any assessment of risk of bias that may affect the cumulative evidence (e.g., publication bias, selective reporting within studies). | 8, Table 1 |
| Additional analyses | 16 | Describe methods of additional analyses (e.g., sensitivity or subgroup analyses, meta-regression), if done, indicating which were pre-specified. | - |
| **RESULTS** | | |  |
| Study selection | 17 | Give numbers of studies screened, assessed for eligibility, and included in the review, with reasons for exclusions at each stage, ideally with a flow diagram. | 7-8  Fig.1 |
| Study characteristics | 18 | For each study, present characteristics for which data were extracted (e.g., study size, PICOS, follow-up period) and provide the citations. | 7-8, Table1 |
| Risk of bias within studies | 19 | Present data on risk of bias of each study and, if available, any outcome level assessment (see item 12). | 6-8, Fig.2 |
| Results of individual studies | 20 | For all outcomes considered (benefits or harms), present, for each study: (a) simple summary data for each intervention group (b) effect estimates and confidence intervals, ideally with a forest plot. | 8-12, Table 2, Figs3-6 |
| Synthesis of results | 21 | Present results of each meta-analysis done, including confidence intervals and measures of consistency. | 8-12, Table 2, Figs3-6 |
| Risk of bias across studies | 22 | Present results of any assessment of risk of bias across studies (see Item 15). | Table 1 |
| Additional analysis | 23 | Give results of additional analyses, if done (e.g., sensitivity or subgroup analyses, meta-regression [see Item 16]). | - |
| **DISCUSSION** | | |  |
| Summary of evidence | 24 | Summarize the main findings including the strength of evidence for each main outcome; consider their relevance to key groups (e.g., healthcare providers, users, and policy makers). | Table GRADE |
| Limitations | 25 | Discuss limitations at study and outcome level (e.g., risk of bias), and at review-level (e.g., incomplete retrieval of identified research, reporting bias). | 13-14 |
| Conclusions | 26 | Provide a general interpretation of the results in the context of other evidence, and implications for future research. | 14 |
| **FUNDING** | | |  |
| Funding | 27 | Describe sources of funding for the systematic review and other support (e.g., supply of data); role of funders for the systematic review. | 14-15 |

*From:*  Moher D, Liberati A, Tetzlaff J, Altman DG, The PRISMA Group (2009). Preferred Reporting Items for Systematic Reviews and Meta-Analyses: The PRISMA Statement. PLoS Med 6(7): e1000097. doi:10.1371/journal.pmed1000097

**Search keys:**

In PubMed, we searched with the following terms: ("diabetes mellitus, type 2"[MeSH Terms] OR "type 2 diabetes mellitus"[All Fields] OR "type 2 diabetes"[All Fields]) AND (("probiotics"[MeSH Terms] OR "probiotics"[All Fields]) OR ("probiotics"[MeSH Terms] OR "probiotics"[All Fields] OR "probiotic"[All Fields]) OR ("lactobacillus"[MeSH Terms] OR "lactobacillus"[All Fields]) OR ("streptococcus"[MeSH Terms] OR "streptococcus"[All Fields]) OR ("saccharomyces"[MeSH Terms] OR "saccharomyces"[All Fields]) OR ("enterococcus"[MeSH Terms] OR "enterococcus"[All Fields]) OR ("bifidobacterium"[MeSH Terms] OR "bifidobacterium"[All Fields])). In the Cochrane Library we used the following search terms: diabetes mellitus type 2 AND (probiotic* OR lactobacillus OR saccharomyces OR enterococcus OR escherichia coli OR streptococcus OR bifidobacterium) AND random* in All Text’. In EMBASE, we searched the following terms: ('diabetes mellitus type 2'/exp OR 'diabetes mellitus type 2' OR (('diabetes'/exp OR diabetes) AND mellitus AND type AND 2)) AND (probiotic* OR 'lactobacillus'/exp OR lactobacillus OR 'saccharomyces'/exp OR saccharomyces OR 'enterococcus'/exp OR enterococcus OR 'escherichia coli'/exp OR 'escherichia coli' OR (('escherichia'/exp OR escherichia) AND coli) OR 'streptococcus'/exp OR streptococcus OR 'bifidobacterium'/exp OR bifidobacterium) AND random*

Supplementary figures:

**Supplementary figure 1. (S1 figure)**

**Supplementary figure 2. (S2 figure)**


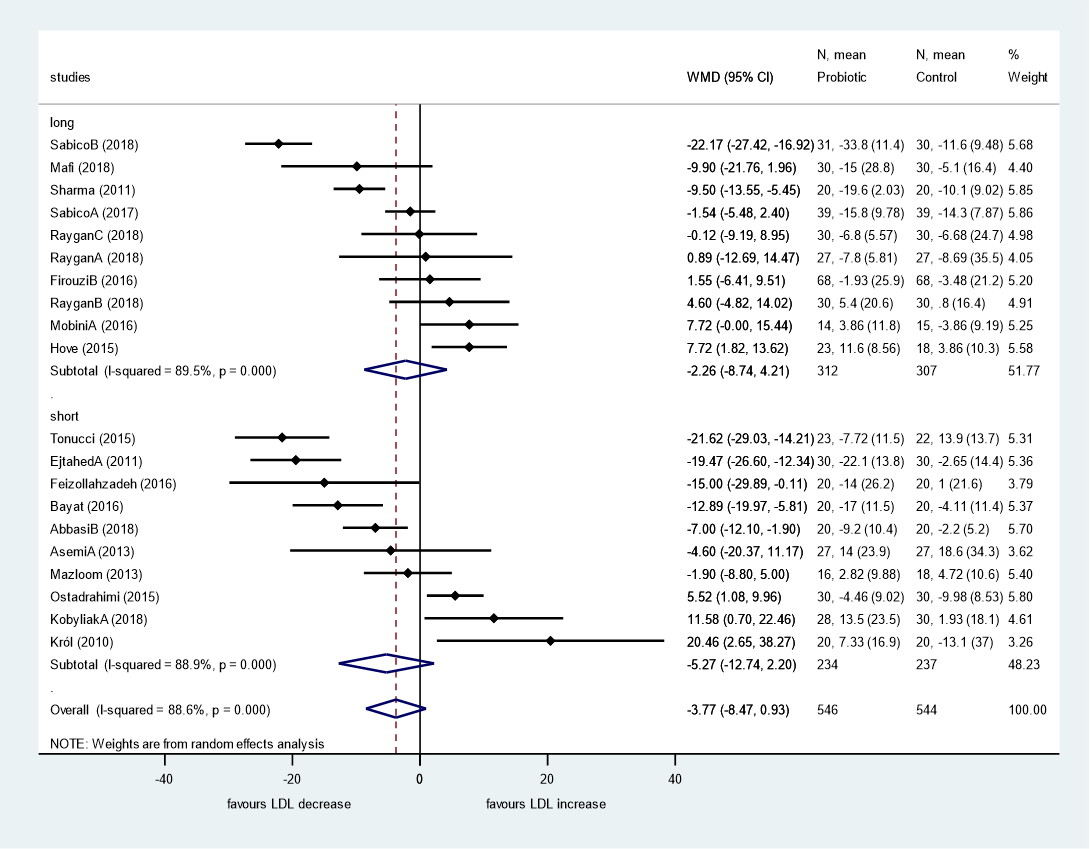


**Supplementary figure 3. (S3 figure)**

**Supplementary figure 4. (S4 figure)**

**Supplementary figure 5. (S5 figure)**

**
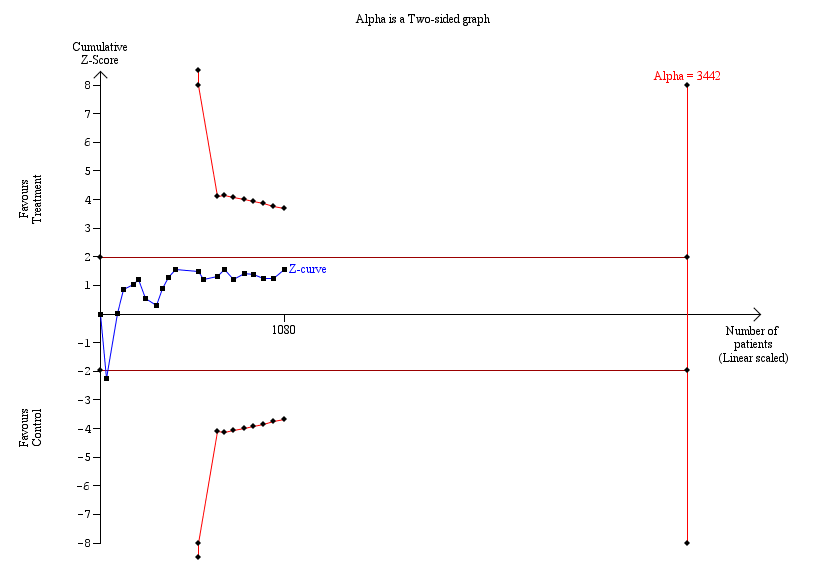
**

**Supplementary figure 6. (S6 figure)**

**Supplementary figure legends:**

**Supplementary figure 1.** Forest plot for the effect of probiotics on total cholesterol (T-chol) compared to controls in pooled analysis. The shaded diamonds indicate the effect of probiotics in a particular study (weighted difference in mean). The horizontal lines represent 95% confidence intervals (CIs). The big diamond data marker indicates the pooled effect. The figure shows the summary of studies overall and subdivided by the number of bacterial species used. “multiple”: combination of bacteria, “single”: one bacterial species used

**Supplementary figure 2.** Forest plot for the effect of probiotics on low density lipoprotein (LDL) compared to controls in pooled analysis. The shaded diamonds indicate the effect of probiotics in a particular study (weighted difference in mean). The horizontal lines represent 95% confidence intervals (CIs). The big diamond data marker indicates the pooled effect. The figure shows the summary of studies overall and subdivided by length of intervention. “long”: 12 weeks or longer, “short”: 8 weeks or shorter.

**Supplementary figure 3.** Forest plot for the effect of probiotics on low density lipoprotein (LDL) compared to controls in pooled analysis. The shaded diamonds indicate the effect of probiotics in a particular study (weighted difference in mean). The horizontal lines represent 95% confidence intervals (CIs). The big diamond data marker indicates the pooled effect. The figure shows the summary of studies overall and subdivided by the number of bacterial species used. “multiple”: combination of bacteria, “single”: one bacterial species used

**Supplementary figure 4.** Forest plot for the effect of probiotics on low density lipoprotein (LDL) compared to controls in pooled analysis. The shaded diamonds indicate the effect of probiotics in a particular study (weighted difference in mean). The horizontal lines represent 95% confidence intervals (CIs). The big diamond data marker indicates the pooled effect. The figure shows the summary of studies overall and subdivided by the number of bacterial species used. “multiple”: combination of bacteria, “single”: one bacterial species used

**Supplementary figure 5.** Trial sequential analysis of data on low density lipoprotein (LDL). Trial sequential analysis is a random effect-based meta-analytical model to estimate the “required information size” i.e. the sample size allowing us to draw a confident conclusion. Each dot on the Z-curve represents a new piece of information, the results of a new randomised study. In our case, the analysis shows that our sample size is insufficient to draw a confident conclusion.

**Supplementary figure 6.** Forest plot for the effect of probiotics on fasting plasma glucose (FPG) compared to controls in pooled analysis. The shaded diamonds indicate the effect of probiotics in a particular study (weighted difference in mean). The horizontal lines represent 95% confidence intervals (CIs). The big diamond data marker indicates the pooled effect. The figure shows the summary of studies overall and subdivided by the number of bacterial species used. “multiple”: combination of bacteria, “single”: one bacterial species used
